# Diagnostic criteria for invasive pulmonary aspergillosis in chronic obstructive pulmonary disease patients

**DOI:** 10.1093/ajrccm/aamag310

**Published:** 2026-07-01

**Authors:** David W Denning, Thomas R Rogers, Takahiro Takazono, Xin Su, Katrien Lagrou, P Lewis White, Darius Armstrong James, Mona Bafadhel, Jose Barbaran Lopez, Pierre Bulpa, Sanjay H Chotirmall, Sara Gago, Jesus Guinea, David M G Halpin, MeiLan K Han, John R Hurst, Sebastien Imbert, Wim Janssens, Chris Kosmidis, Alexander G Mathioudakis, Animesh Ray, Nicolas Roche, Yongchang Sun, Pei Yee Tiew, Claus F Vogelmeier, Thomas M Wilkinson, Jørgen Vestbo

**Affiliations:** Manchester Fungal Infection Group, Faculty of Biology, Medicine and Health, The University of Manchester, Manchester Academic Health Science Centre, Manchester, United Kingdom; Department of Clinical Microbiology, Trinity College Dublin, St. James’s Hospital Campus, Dublin, Ireland; Department of Infectious Diseases, Nagasaki University Graduate School of Biomedical Sciences, Nagasaki, Japan; Department of Respiratory and Critical Medicine, Nanjing Drum Tower Hospital, Affiliated Hospital of Medical School, Nanjing University, Nanjing, China; Department of Microbiology, Immunology and Transplantation, KU Leuven, Leuven, Belgium; Department of Laboratory Medicine and National Reference Center for Mycosis, University Hospitals Leuven, Leuven, Belgium; Public Health Wales Mycology Reference Laboratory, University Hospital of Wales, Cardiff, United Kingdom; Centre for Trials Research, Cardiff University, University Hospital of Wales, Cardiff, United Kingdom; Department of Infectious Diseases, Imperial College, University of London, London, United Kingdom; King’s Centre for Lung Health, School of Immunology and Microbial Sciences, King’s College London, London, United Kingdom; HM Hospitals Health Research Institute, University Center for Health Sciences—HM Hospitals (CUHMED), Camilo Jose Cela University, Madrid, Spain; Service des Soins Intensifs, Mont-Godinne University Hospital, CHU UCL Namur, Namur, Belgium; Lee Kong Chian School of Medicine, Nanyang Technological University, Singapore; Department of Respiratory and Critical Care Medicine, Tan Tock Seng Hospital, Singapore; Manchester Fungal Infection Group, Faculty of Biology, Medicine and Health, The University of Manchester, Manchester Academic Health Science Centre, Manchester, United Kingdom; Clinical Microbiology and Infectious Diseases Department, Hospital General Universitario Gregorio Marañón, Madrid, Spain; Instituto de Investigación Sanitaria Gregorio Marañón, Madrid, Spain; CIBER Enfermedades Respiratorias (CIBERES), Madrid, Spain; Faculty of Health Sciences—HM Hospitals, Universidad Camilo José Cela, Madrid, Spain; College of Medicine and Health, University of Exeter Medical School, University of Exeter, Exeter, United Kingdom; Division of Pulmonary and Critical Care, University of Michigan, Ann Arbor, MI, United States; UCL Respiratory, University College London, London, United Kingdom; Centre de Recherche Cardio-Thoracique de Bordeaux, INSERM U1045, Universite de Bordeaux, Pessac, France; Service de Parasitologie-Mycologie, CHU Bordeaux, Bordeaux, France; Sydney Infectious Diseases Institute, The University of Sydney, Sydney, Australia; Respiratory Division, University Hospitals Leuven, Leuven, Belgium; CHROMETA Department, KU Leuven, Leuven, Belgium; Manchester Fungal Infection Group, Faculty of Biology, Medicine and Health, The University of Manchester, Manchester Academic Health Science Centre, Manchester, United Kingdom; National Aspergillosis Centre, Department of Infectious Diseases, Manchester University NHS Foundation Trust, Manchester, United Kingdom; Division of Immunology, Immunity to Infection and Respiratory Medicine, School of Biological Sciences, Faculty of Biology, Medicine and Health, The University of Manchester, Manchester, United Kingdom; The North West Lung Centre, Wythenshawe Hospital, Manchester University NHS Foundation Trust, Manchester Academic Health Science Centre, Manchester, United Kingdom; Department of Medicine, All India Institute of Medical Sciences, New Delhi, India; Service de Pneumologie, AP-HP Centre, Université Paris Cité, Hôpital et Institut Cochin, Paris, France; Department of Respiratory and Critical Care Medicine, Peking University Third Hospital, Beijing, China; Lee Kong Chian School of Medicine, Nanyang Technological University, Singapore; Department of Respiratory and Critical Care Medicine, Singapore General Hospital, Singapore; Department of Medicine, Pulmonary and Critical Care Medicine, Philipps University of Marburg, German Center for Lung Research (DZL), Marburg, Germany; Faculty of Medicine, University of Southampton, Southampton, United Kingdom; The North West Lung Centre, Wythenshawe Hospital, Manchester University NHS Foundation Trust, Manchester Academic Health Science Centre, Manchester, United Kingdom

**Keywords:** antifungal therapy, CT scan, cavitation, stewardship, diagnostic performance, tracheobronchitis

## Abstract

More than 400 million people have chronic obstructive pulmonary disease (COPD), with exacerbations representing a major health burden. Although the overall incidence of invasive pulmonary aspergillosis (IPA) in patients with COPD with hospitalized exacerbation is only 1% to 4%, certain factors substantially increase this frequency. Risk factors compromising defenses against *Aspergillus* spp. in COPD patients include systemic or high-dose inhaled corticosteroids; comorbidities including bronchiectasis, diabetes, and cardiovascular disease; and prolonged courses of antibiotics. An international group of experts met to develop criteria for diagnosing IPA based on existing literature and consensus in nonventilated COPD patients. The preliminary diagnostic recommendations were further evaluated by additional experts using the Delphi methodology. A hospitalized exacerbation of COPD with 2 or more of the above clinical risk factors should prompt (1) a computed tomography scan of the chest; (2) sending a respiratory sample (sputum, induced sputum or bronchoscopy sample) for direct microscopy for fungi, high-volume fungal culture, and preferably *Aspergillus* PCR and, if a bronchoscopy sample is obtained, then also *Aspergillus* antigen (galactomannan); and (3) a serum sample for galactomannan and *Aspergillus* IgG. The combination of a high-risk COPD patient, with compatible imaging abnormalities and any 2 positive tests (2 samples or different tests on the same respiratory sample) for *Aspergillus* is sufficient to establish the diagnosis of IPA with enough confidence to initiate antifungal therapy and/or enroll the patient in a clinical or epidemiological study of IPA in COPD. Additional studies are required to augment performance data for most assays in COPD and validate the proposed diagnostic criteria.

## Introduction

Since it was first described in 1953, invasive pulmonary aspergillosis (IPA) has been increasingly recognized in immunocompromised patients. IPA is defined as *Aspergillus* hyphae invading tissue; however, as this is rarely documented in clinical practice, alternative means of establishing an early diagnosis are now usually used. The introduction of computed tomography (CT) scanning in the 1980s and descriptions of the characteristic features of the “halo” sign and cavitation were pivotal in enabling faster diagnosis and therapy in neutropenic leukemia patients.^1^ Then the discovery and commercialization of the galactomannan (GM) antigen for more precise serum-based diagnosis was pivotal for leukemia patients with neutropenia, but less so other patient groups.^2-4^ Once GM antigen detection in bronchoalveolar lavage fluid (BAL) became mainstream for a broader patient population, the diagnosis of IPA was transformed. BAL GM is substantially more sensitive than culture as a biomarker for IPA diagnosis. The use of CT scanning and GM detection were codified in the first and subsequent iterations of the European Organisation for research and treatment of Cancer (EORTC)/Mycoses Study Group Education and Research Consortium (MSGERC) definitions of IPA for immunocompromised patients,^5-7^ but excluded those in ICUs or for those with chronic obstructive pulmonary disease (COPD) as not classically immunocompromised.

The incidence of IPA in prospective, retrospective, and insurance claim studies in patients admitted to hospital with COPD varies from 0.1% in the United States to 3.9% in China.^8-12^ A key feature of IPA in COPD is that it primarily occurs in those with COPD exacerbations. These are frequent in COPD; many patients experience multiple exacerbations within a year, posing a significant burden to the health system.^13^ Exacerbations are mainly treated with antibiotics and/or systemic corticosteroids and these 2 factors may in themselves contribute to development of IPA. Given the large number of patients, IPA requires earlier clinical consideration than is currently the case in most centers, to reduce unnecessary and possibly harmful antibacterial and corticosteroid therapy, ICU admission, prolonged hospital stays, and mortality. At the same time, there is a need to limit unnecessary antifungal treatment, again to reduce harm, costs, and risk of antifungal resistance.

Prior consensus definitions of IPA in immunocompromised and ICU patients have relied heavily on bronchoscopic mycological findings, which is often not possible or not done as an emergency in COPD patients hospitalized with an exacerbation. A prior single-center study of IPA in COPD patients proposed definitions nearly 20 years ago,^14^ prior to the advent of *Aspergillus* PCR, study, and validation of serum *Aspergillus* IgG assays, and before several studies of IPA in COPD patients were published delineating specific COPD risk factors. Sputum is often obtainable in such patients, but not usually processed thoroughly enough to detect *Aspergillus* spp. Imaging findings on CT scans are typically not as distinctive as in neutropenic patients, and often not done until late in the course of hospitalization. COPD patients are very heterogenous in terms of IPA risk, hence the need to derive updated and consensus definitions.

While sustained systemic corticosteroids in those with COPD have been recognized as a significant risk factor for IPA,^14-16^ many cases are described in those not taking systemic corticosteroids.^9^^,^^17^ Furthermore, cumulative dose of oral corticosteroid is a significant risk factor.^8^^,^^9^^,^^18^ Antibacterial use^9^^,^^10^ and inhaled corticosteroids may also contribute,^19^ as may nosocomial transmission.^18^^,^^20^ Global Initiative for Chronic Obstructive Lung Disease (GOLD) stage III or IV of COPD is linked to IPA in some studies,^8^ but not others.^21^ Other factors associated with IPA are heart disease, diabetes, bullae, and bronchiectasis.^12^^,^^21^

Here we set out expanded consensus diagnostic criteria for IPA in patients with COPD exacerbations admitted to hospital. One key benefit of earlier and accurate diagnosis of IPA could be avoidance of ICU admission. We do not address stable COPD patients, nor do we address intubated COPD patients, as latter patients have been addressed recently in 2 separate guidelines.^22^^,^^23^ We also do not provide a new or different definition for proven IPA in COPD as this has been previously well described.^5-7^^,^^14^ The focus is patients with COPD admitted to hospital with an exacerbation, including patients needing noninvasive ventilation, as recently set out in the updated 2026 GOLD guidelines.^13^

## Methods

A group of international experts in respiratory medicine with a focus on COPD, infectious diseases, and medical mycology was assembled to evaluate key topics relevant to developing a consensus definition of IPA in COPD patients, using published data. They first focused on narrowing down the risk profile of all COPD patients to those with a higher probability of IPA, so that any diagnostic tests would build on a higher pretest probability enhancing the likelihood of disease if positive. The second focus was the specificity of radiological features of IPA in COPD. The third focus was on the diagnostic performance of microscopy, fungal culture, *Aspergillus* antigen (GM), *Aspergillus* PCR, and next-generation sequencing (NGS) on respiratory specimens, whether obtained by bronchoscopy or in sputum. The fourth area focused on serum assays: GM, *Aspergillus* antibody, and 1,3-β-D-glucan (BDG). For each area, a scoping review approach was taken to the literature, given the heterogeneity of the literature and limited availability of formal, prospective studies in this patient population.

The workshop convened on December 4, 2025, in Manchester, United Kingdom. Nominated speakers summarized the literature in specific domains (as above), and extensive discussion followed. The 4 domains were gradually built up by consensus, providing a structured approach to suspecting IPA, ruling it in or out with imaging, distinguishing *Aspergillus* colonization from *Aspergillus* tracheobronchitis and IPA, and recommending what additional tests to do to confirm or refute a diagnosis of IPA.

During the meeting, contributions in each domain were summarized for review by the group, and the precise wording was discussed and decided upon. The proposed diagnostic criteria were then presented to a larger audience of stakeholders.

Following the consensus meeting, an additional step of consensus building was achieved with 2 rounds of Delphi online questions.^24^ Experts were split into clinical and laboratory groups to utilize the respective knowledge bases of the participants, and additional clinical expertise was drawn from those invited to the meeting but unable to attend. The Delphi tool was provided by Information Management Associates (https://www.informat.org/delphi/). All uncertainties or areas of nonagreement were formulated as questions (see supplementary information 1). Round 1 of Delphi questions enabled majority opinions (with a 75% threshold) to be incorporated into the final consensus. The final criteria were assembled and participants were encouraged to identify and add any additional supportive or contrary data. Two rounds of review were made.

As no new patient data were considered, ethics review was not required.

## Results

### Clinical features of IPA and tracheobronchitis in COPD

It is not possible to make a diagnosis of IPA or invasive *Aspergillus* tracheobronchitis based on clinical symptoms and signs alone, even with apparently characteristic but no pathognomonic radiological features; the differential diagnosis is wide and most patients (especially those with early disease) have no new symptoms attributable to aspergillosis. Fever is unusual. Pointers to the diagnosis include hemoptysis (uncommon), chest ache or discomfort, and new wheeze, any of which, if present, should prompt urgent further investigation, especially if other risk factors are also present. Even distinguishing IPA from *Aspergillus* tracheobronchitis is challenging, and they may occur together.

Acute inflammatory markers are usually raised in IPA in non-immunocompromised patients.^25^ Therefore, a normal C-reactive protein would indicate a low chance of IPA,^25^ but this would be unusual in the context of a COPD exacerbation. D-dimers are often measured in severe COPD exacerbations as pulmonary embolism is an important differential diagnosis. IPA is characterized by angioinvasion, pulmonary infarction, and similar appearances on CT scan to pulmonary emboli. D-dimers are raised in IPA.^26^ Therefore, a diagnosis of pulmonary emboli in COPD should prompt urgent consideration of IPA.

### COPD risk assessment for suspected IPA and *Aspergillus* tracheobronchitis

**Figure 1:**
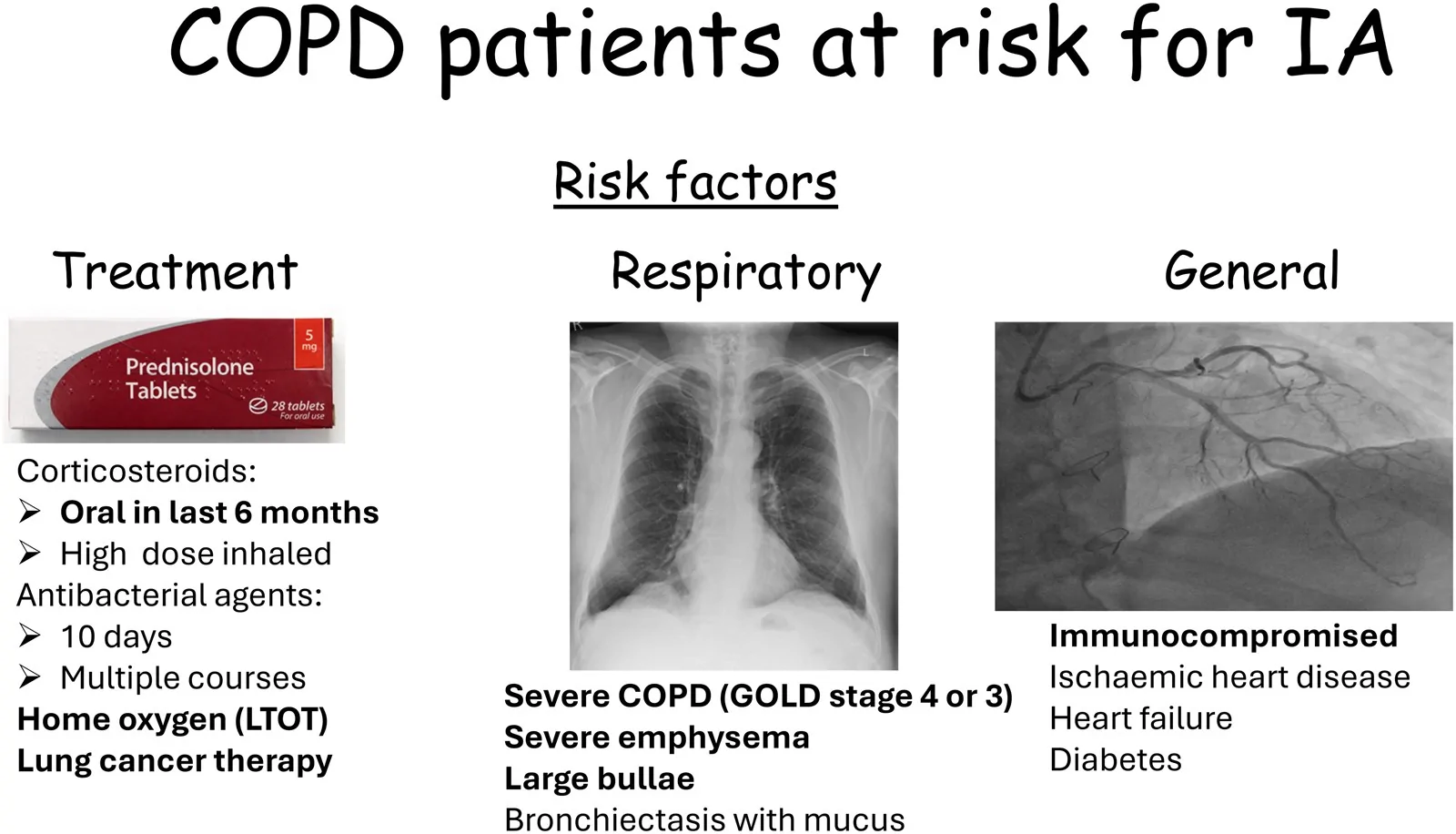
Risk factors associated with IPA in COPD patients. Bold text indicates the highest-risk factors or associations with IPA. Abbreviations: COPD, chronic obstructive pulmonary disease; GOLD, Global Initiative for Chronic Obstructive Lung Disease; IA, invasive aspergillosis; IPA, invasive pulmonary aspergillosis; LTOT, long-term oxygen therapy. See Table 1 for weighing of risk factors.

The ranking of risk factors from the Delphi questionnaire is shown in Table 1, the supplementary file, and Figure 1.

**Table 1:** Risk factors for IPA in admitted patients with a COPD exacerbation, listed in decreasing order of risk (Delphi responses).

**1 (Highest)**	Major immunocompromising factor Oral corticosteroids: for >10 days > oral corticosteroid for >5 days > any oral corticosteroid in the last 3 months Severe emphysema GOLD stage 4
**2**	GOLD stage 3 Home oxygen requirement Lung cancer treatment High-dose inhaled corticosteroids Large bullae
**3**	Cardiac failure or ischemic heart disease Prior exacerbation in the last 12 months Diabetes Bronchiectasis with mucus production
**4 (Lowest)**	Clinically inactive or radiological bronchiectasis only Low-dose inhaled corticosteroids GOLD stage 1 or 2

No question was asked about antibacterial therapy, which is a documented risk factor for IPA in some studies. Neither was a question asked about medium-dose inhaled corticosteroids, which have recently emerged as a moderate risk factor for IPA.

Abbreviations: COPD, chronic obstructive pulmonary disease; GOLD, Global Initiative for Chronic Obstructive Lung Disease; IPA, invasive pulmonary aspergillosis.

### Radiological features of IPA and *Aspergillus* tracheobronchitis in COPD

Consensus was for performing a contrast-enhanced CT scan in COPD, as it includes the documentation and severity evaluation of bronchiectasis, documentation of prior tuberculosis (with its own sequelae/endotype of COPD), suspicion of chronic pulmonary aspergillosis (CPA) (usually cavitation, with or without a fungal ball), assessment of emphysema and/or bullae, ruling out a lung tumor or other infection such as histoplasmosis or coccidioidomycosis, and evaluation for invasive pulmonary or tracheobronchial aspergillosis. If a CT scan has been done recently, some of these differential or additional diagnoses will already have been ruled in or out; however, low-dose CT as often applied for screening for lung cancer is not sensitive enough to rule out IPA.

Chest X-rays are useful if new abnormalities suggestive of IPA are seen; this finding should prompt an urgent chest CT scan. A normal chest X-ray does not exclude IPA^9^^,^^27^ for several reasons: small nodules may be missed, airways abnormalities are not seen and findings may be obscured behind the heart or mediastinal structures or preexisting abnormalities. In a series of 26 COPD patients with IPA, the initial chest X-ray was normal in 15 (58%); however, new or progressive infiltrates were found in 17 of 30 (65%) chest X-rays when repeated.^9^

While CT findings are not highly specific for IPA, 2 patterns emerge—one set angioinvasive, the second set airway invasive.^28^ Airway invasion changes may represent an earlier stage of disease, preceding angioinvasive features in many cases of IPA. A retrospective multicenter study in China including 57 (22.4%) COPD patients, of whom 18% were ventilated, showed the following airway invasion features: patches distributed along one or more airways (68%), centrilobular nodules (37%), tree-in-bud sign (40%) thickened airways (42%), pleural effusion (28%), and atelectasis (6%). In the same study, angioinvasive findings included cavitation (38%), a soft tissue mass (30%), halo sign (19%), an air crescent sign (11%), and wedge-shaped consolidation (7%). Another study of 72 histology-confirmed IPA cases, of whom 28 (39%) had COPD, found a variety of patterns including 27 (37.5%) patients with radiological bronchiectasis and 22 (30.6%) with peribronchial consolidation; in addition, 18 (25.0%) had nodules and/or patchy shadows with small cavities peripherally.^29^ The specificity of CT findings has not been systematically addressed but may be low^9^; angioinvasion is also found in severe COVID-19 pneumonitis, for example, with nodules and halo signs.

Findings of cavitation (including an air crescent sign) or a halo sign were regarded as more likely to represent IPA than other features seen on CT. None of the airway features seen in invasive *Aspergillus* tracheobronchitis were thought to be specific enough to diagnose IPA with confidence, and their absence does not exclude it.

### Sputum, bronchoscopy, and induced sputum

Consensus was that respiratory samples are required to confirm IPA or tracheobronchial aspergillosis, or to substantially reduce its likelihood if all are negative.

In awake patients, spontaneous or induced sputum or bronchoscopy with bronchial wash/BAL should yield diagnostic specimens. The sensitivity of *Aspergillus* PCR is similar or better than fungal culture from induced sputum specimens or those obtained using active cycle of breathing techniques.^30^ Induced sputum can be done on COPD patients safely if carefully supervised, even during exacerbations, as long as baseline FEV_1_ is >30% at baseline.^31^^,^^32^ Typically, 5% to 7% hypertonic saline is used; in very breathless patients some units use normal saline with reasonable sputum sample quality and less risk of severe bronchospasm.^33^ In all cases, induction is preceded by inhaled salbutamol, and for this reason those with significant risk of cardiac arrhythmias may be excluded from sputum induction. Sputum obtained by induction should be processed for direct microscopy, fungal culture, and commercial or validated in-house *Aspergillus* PCR, if the latter is available.

Bronchoscopy and bronchial wash/BAL in patients with an acute exacerbation of COPD is not often conducted but could yield useful diagnostic information, notably *Aspergillus* antigen and visual confirmation of tracheobronchial aspergillosis, seeing airway plaques, hemorrhage, and/or ulceration. The risks and benefits should be considered on an individual patient basis. Bronchoscopy to collect diagnostic samples should be strongly considered in patients who are at risk of IPA and are not responding to optimal therapy for a COPD exacerbation. If bronchial or tracheal lesions are seen, a biopsy for histopathology, direct microscopy, and culture as well as *Aspergillus* antigen are strongly recommended.

### Respiratory sample diagnostics

Consensus views are presented; more details on sample processing and each test are provided in the online supplement.

#### Direct microscopy

The consensus view was that direct microscopy should be done on all samples submitted for fungal culture in this cohort of at-risk patients.^34^ A positive result will be fast and helpful (very high specificity for aspergillosis, usually invasive); however, a negative result does not rule out any form of aspergillosis. A positive direct microscopy showing branching fungal hyphae is proportionately more common in those with airway aspergillosis (ie, invasive *Aspergillus* tracheobronchitis or *Aspergillus* bronchitis), but may also be positive in those with IPA and CPA.

The value of direct microscopy is its low cost and simplicity, immediacy, and high utility if positive. The downside of direct microscopy is that it requires considerable skills to interpret (eg, true hyphae can be mistaken for pseudohyphae of *Candida* spp.) and it takes technical time (especially if nonfluorescent methods are used). Data are lacking on the sensitivity of direct microscopy for both IPA and invasive tracheobronchitis in COPD patients, but it is likely to be ≪50% based on other groups of patients.

#### Fungal culture

The consensus was that every effort should be made to send at least one, and preferably 3, respiratory samples for fungal culture from those at risk of IPA. Expectorated or induced sputum, bronchoscopy samples, sputum after bronchoscopy, or aspiration with coughing and sputum production are all potentially valuable in facilitating a diagnosis of IPA.

A negative culture does not rule out IPA, as sensitivity is low. A positive culture may reflect colonization, IPA, invasive *Aspergillus* tracheobronchitis, CPA, or *Aspergillus* bronchitis. Thus, interpretation requires the clinical context and fungal biomarkers or typical radiology to confidently diagnose IPA.

The yield of fungi from bacterial culture plates alone is about 30% lower than when fungal media are used.^35^ Sensitivity can also be increased by using high-volume culture techniques.^36^ Multiple specimens for culture increases diagnostic sensitivity, as for all forms of pulmonary aspergillosis, although not assessed in COPD patients. A positive culture allows species identification, which has some implications for antifungal choice (eg, *Aspergillus terreus* is resistant to amphotericin B). Susceptibility testing is also possible, to detect azole resistance.

Triazole therapy is markedly superior to therapy with echinocandins or amphotericin B in reducing mortality from IPA.^37^^,^^38^ However, triazole resistance in *Aspergillus fumigatus*, *Aspergillus niger* complex, and *Aspergillus flavus* is now commonplace in environmental and some clinical isolates.^39-41^ There is a strong correlation between clinical treatment failure and azole resistance in *Aspergillus* spp. Therefore, susceptibility testing of *Aspergillus* against the triazoles is advantageous, benefiting the patient being treated and also deriving data useful to direct empirical therapy, as is done for pathogenic bacteria. Optimizing culture yield is therefore important for this reason alone.

#### Aspergillus GM antigen

The consensus was that sputum GM assays do not provide sufficient data for confirming or excluding a diagnosis of IPA in COPD patients. On the other hand, BAL GM is a very valuable test for diagnosing IPA in most patient groups, but may be positive in CPA or falsely positive.

GM is a major *Aspergillus* cell wall antigen released from the tip of growing fungal hyphae. It has become established as a fungal biomarker for diagnosis of IPA in immunocompromised patients, especially those with profound neutropenia. Its diagnostic role is less well validated in non-immunocompromised persons, including nonventilated COPD patients.

GM as a diagnostic biomarker in COPD patients with suspected IPA has been assessed in only a limited number of studies investigating BAL (Table 2). In 2 studies of hospitalized COPD patients with suspected IPA,^42^^,^^46^ BAL GM had better sensitivity than serum/plasma GM, but overall showed poorer performance than documented in studies of GM in immunocompromised patients. In a prospective study of COPD patients with suspected IPA where samples were taken from the day of admission to ICU, BAL GM again performed better than serum GM, and both assays in this study had better sensitivity and specificity than in the earlier cited studies, possibly due to these patients having more advanced COPD and/or IPA.^46^ In COPD patients with IPA BAL GM was 86.7% sensitive in 26 clinically suspected cases^47^ (Figure 3).

**Table 2:** Performance of biomarkers and *Aspergillus* IgG antibody for the diagnosis of IPA in COPD patients.

Patients	Study design	Method	Optical density cutoff	Sensitivity	Specificity	Reference
**Serum GM (*Aspergillus* antigen)**						
** Hospitalized COPD patients with suspected IPA**	Retrospective multicenter cohort study including COPD (*n* = 35)	Platelia EIA, Sanofi Diagnostics,	0.5	11.1%	96.2%	Fortún et al., 2016^44^
** Patients with stable COPD (*n*** = **191)**	Retrospective study—link between serum GM and acute exacerbations of COPD	Platelia EIA	0.5	NS GM positivity (ODI >0.7) predicted severe AE-COPD and reduced survival	NS	Yoshimura et al., 2018^45^
** Hospitalized nonneutropenic patients with suspected IPA including COPD (14/41)**	Multicenter retrospective (proven IPA *n* = 10; putative IPA *n* = 31)	Platelia EIA	0.5	42.9%	90.3%	Dobias et al., 2018^46^
** Patients with IPA (*n*** = **37)**	Diagnostic comparison with healthy control (*n* = 50) and bacterial pneumonia (*n* = 15)	Dynamiker ELISA GM	≥0.85 μg/L	29.7%	NS	Yu et al, 2020^47^
** Hospitalized COPD patients admitted with suspected IPA (*n*** = **165)**	Retrospective case-control study of IPA (*n* = 35) vs no IPA (130)	Platelia EIA	0.55	60%	73.8%	He et al., 2021^48^
** Hospitalized nonneutropenic patients with suspected IPA including COPD (76/368)^a^**	Multicenter, prospective, study (proven IPA *n* = 10; probable IPA *n* = 75; possible IPA *n* = 14)	Platelia EIA	1.0	19.2%	96.3%	Lu et al., 2023^49^
** Hospitalized patients with acute exacerbations of COPD (*n*** = **76)**	Case control: IPA (*n* = 39) vs non-IPA (*n* = 37)	Shanghai Fanwei Biotech	0.5	64.1%	75.7%	Hu et al., 2024^50^
** Patients attending the ED with acute exacerbations of COPD (*n*** = **61)**	Cross-sectional	Platelia EIA	0.5	44.4% (Positivity associated with hemoptysis and prior TB)	83.7%	Palanivel et al., 2025^51^
**Serum *Aspergillus* IgG antibody**						
** Hospitalized nonneutropenic patients with suspected IPA including COPD (14/41)**	Multicenter retrospective (proven IPA *n* = 10; putative IPA *n* = 31)	Immunolab ELISA IgG/IgA	≥1.2	96.4%	93.5%	Dobias et al., 2018^46^
** Patients with IPA (*n*** = **37) or CPA (*n*** = **21)**	Diagnostic comparison with healthy control (*n* = 50) and bacterial pneumonia (*n* = 15)	Dynamiker ELISA IgG	≥120 AU/mL	45.9%	92.3%	Yu et al., 2020^47^
** Hospitalized nonneutropenic patients with suspected IPA including COPD (76/368)^a^**	Multicenter, prospective, study (proven IPA *n* = 10; probable IPA *n* = 75; possible IPA *n* = 14)	Dynamiker ELISA IgG	≥120 AU/mL	59.6%	77%	Lu et al., 2023^49^
** Patients with suspected IPA without immunocompromise or end-stage COPD (20/59 had COPD)**	Prospective, single-center controlled (proven IPA *n* = 7; probable IPA *n* = 43; possible IPA *n* = 9)	Dynamiker ELISA IgG	≥120 AU/mL^b^	54.2%	89.7%	Sheng et al., 2024^52^
**Serum BDG**						
** Hospitalized nonneutropenic patients with suspected IPA including COPD (14/41)**	Multicenter retrospective (proven IPA *n* = 10; putative IPA *n* = 31)	Fungitell EIA	≥80 pg/mL	82.9%^c^	73.9%	Dobias et al., 2018^46^
** Patients with IPA (*n*** = **37)**	Diagnostic comparison with healthy control (*n* = 50) and bacterial pneumonia (*n* = 15)	Dynamiker ELISA	≥120 pg/mL	32.4%^c^	NS	Yu et al., 2020^47^
**BAL fluid GM (*Aspergillus* antigen)**						
** Hospitalized COPD patients with suspected IPA**	Retrospective multicenter cohort study including COPD (*n* = 35)	Platelia EIA	1.0	66.7%	96.2%	Fortún et al., 2016^44^
** Hospitalized COPD patients admitted with suspected IPA (*n*** = **165)**	Retrospective case-control study of IPA (*n* = 35) vs no IPA (130)	Platelia EIA	0.8	78.6%	80.7%	He et al., 2021^48^

Papers that included COPD patients predominantly in ICU or with other major risk factors such as neutropenia or which studied CPA are excluded in this table, but included are papers studying nonneutropenic patients with some COPD patients included. The assay used in each study may have been part of the diagnostic criteria for IPA, and each study used different diagnostic criteria for IPA, including for differentiation between probable and possible IPA.

a 101 of 368 (27.4%) were admitted to ICU, including 36 of 99 IPA patients.

b A lower cutoff of 91 AU/mL gave a sensitivity and specificity of 69.5% and 83.8%, respectively.

c Multiple samples were collected and any positive is counted as a positive.

Abbreviations: AE-COPD, acute exacerbation of chronic obstructive pulmonary disease; BDG, 1,3-β-D-glucan; COPD, chronic obstructive pulmonary disease; CPA, chronic pulmonary aspergillosis; ED, emergency department; EIA, enzyme immunoassay; ELISA, enzyme-linked immunosorbent assay; GM, galactomannan; IPA, invasive pulmonary aspergillosis; NS, not stated or not studied; ODI, optical density index; TB, pulmonary tuberculosis.

The limited published data reporting sputum GM in COPD and more generally for other infections are summarized in supplementary information 1.

#### Aspergillus PCR

The consensus was that *Aspergillus* PCR from respiratory samples is an alternative to culture in patients with COPD. (It is not recommended in serum or blood in this patient group, because of a lack of performance data and the potential risk of false positives if not handled cautiously.)

The diagnostic performance of *Aspergillus* PCR versus culture in lower respiratory tract samples from patients with COPD exclusively has been evaluated in 3 studies.^51-53^ Two studies convincingly showed higher yields of PCR than conventional culture in outpatients and hospitalized patients^53^ and one in ICU,^51^ whereas one did not.^52^ The largest and most recent study found 4 of 5 (80%) probable IPA cases to have a positive PCR, compared with 1 of 5 (20%) with a positive culture.^53^ Overall, in 150 sampled COPD patients, PCR was positive in 13 (8.7%), compared to 5 (3.3%) with a positive culture, although most were outpatients, without features suggestive of IPA. Compared with time to positive fungal culture of 3 days, PCR significantly reduced the time to diagnosis to as little as 4 hours using *Aspergillus* PCR.^52^ In Madrid, 91 COPD patients were studied, of whom 6 had probable IPA.^52^ The sensitivity of PCR and culture were excellent at 100% and specificity was 85.9% for PCR and 77.6% for *Aspergillus* culture.

Although reported sensitivity of the *Aspergillus* DNA detection in the respiratory tract was excellent, the mere presence of *Aspergillus* does not necessarily indicate invasive aspergillosis, as is true for culture. Furthermore, different sample types, extraction processes, and PCR kits used partially confound interpretation. Manual bead beating–based extraction produced higher yields than one older automated extraction system, but takes slightly longer in the laboratory.^54^  *Aspergillus* PCR provides complementary data to culture and is often more sensitive.

Some *Aspergillus* PCR systems incorporate species detection and/or markers for drug resistance mutations in the assay,^55-57^ which is faster than conventional susceptibility testing but incomplete in terms of covering all the mechanisms of resistance, and negative PCR reactions do not rule out resistance.

#### NGS detection of *Aspergillus*

The consensus was that NGS detection of *Aspergillus* spp. provided supportive data for IPA if positive, but should only be done on bronchoscopy specimens.

Most studies employed BAL fluid (BALF) samples for metagenomic NGS–based (mNGS) detection of *Aspergillus*, and BALF is additionally endorsed as the preferred sample type for mNGS assays in clinical practice guidelines.^58^ mNGS analysis of BALF has shown higher sensitivity than culture and microscopy and GM in some patient populations, while retaining specificity that is comparable to that of GM detection or targeted PCR.^59-62^ While data specific to the COPD population remain limited, emerging evidence suggests that mNGS can detect *Aspergillus* in COPD patients experiencing acute exacerbations in ICU.^63^ Importantly, mNGS can provide species-level resolution, which is critical given the variable antifungal susceptibility profiles across *Aspergillus* species.^64^ Different approaches to mNGS such as targeted amplicon sequencing have not been directly compared in the context of suspected IPA.

The interpretation of NGS results requires rigorous clinical correlation. Detection of *Aspergillus* DNA does not inherently confirm invasive disease, as colonization or environmental contamination can also lead to positive results.^59^^,^  ^65^ Quantitative metrics (eg, read depth, relative abundance) and integration with diagnostic markers (eg, GM or anti-*Aspergillus* IgG) may enhance diagnostic specificity.^65^^,^^66^ Furthermore, the absence of standardized protocols for sample processing, DNA extraction, sequencing depth, and bioinformatic thresholds currently limits widespread clinical implementation.^64^

### Serum diagnostics

#### Galactomannan

The consensus was that while serum GM is frequently insensitive, and single assays with a slightly raised optical density index may be false positive, dual positives or high-level positives are tantamount to a diagnosis of IPA in COPD, often carrying a poor prognosis (Table 2). Serum GM should be tested on COPD patients suspected of IPA.

Serum GM sensitivity varied from 11.1% to 64%, with considerable variation in diagnostic criteria used and most studies including IPA patients without COPD (Table 2). Specificity varied from 73.8% to 97.8%, but the control groups varied. Positive thresholds also varied and 3 different commercial assays were used.

#### Aspergillus IgG antibody

The consensus was that *Aspergillus* IgG remains a valuable adjunct in the diagnosis of nonneutropenic and COPD-associated IPA, particularly when sufficient time has elapsed for an antibody response and local epidemiology is dominated by *A. fumigatus*. In practice, combining IgG with GM and/or respiratory sample fungal data increases sensitivity and specificity for diagnosis of IPA in COPD. Another consensus was that *Aspergillus*-specific IgE antibody contributes little to a diagnosis of IPA, but data are lacking.

*Aspergillus*-specific IgG has been established as the key serological marker for CPA, with multiple cohorts showing high sensitivity and specificity, and it therefore forms the serologic “anchor” for the chronic end of the disease spectrum (Figure 3). However, when the disease course becomes more acute and shifts toward subacute invasive aspergillosis and IPA, the diagnostic performance of *Aspergillus* IgG is noticeably reduced and much more heterogeneous (Table 2). The Dynamiker anti-GM IgG assay has been evaluated in 4 studies in China with sensitivity ranging from 45.9% to 60.3% and specificity from 77% to 100%. Different patient cohorts were studied, some with IPA, including subacute IPA, with different diagnostic criteria and comparative assays. An earlier study using the Immunolab IgG assay found a 96.4% sensitivity and 93.5% specificity.^44^ Recently a new *Aspergillus* IgG lateral flow assay has been introduced and studied in nonneutropenic patients with acceptable performance^67^ (Table 2).

Importantly, both Lu’s group and subsequent summaries of their multicenter study have emphasized that sensitivity improves with longer symptom duration, with IgG testing showing the greatest diagnostic contribution once the disease course exceeds roughly 10 days in nonneutropenic IPA.^67^ At the same time, several evaluations have shown that some commercial IgG assays targeting only *A. fumigatus* have reduced sensitivity for non-*fumigatus* species, which becomes clinically relevant in regions where *A. flavus*, *A. niger*, or *A. terreus* are prevalent.

#### 1,3-β-D-glucan

The consensus was that BDG may provide supportive data for IPA in COPD, but cannot rule the diagnosis out or in, and more performance data are needed (Table 2). BDG is not specific for aspergillosis, notably being positive in samples from patients with *Pneumocystis* pneumonia or invasive candidiasis, and may sometimes be positive in patients with CPA as well.

Use of Fungitell assay in a small number of patients with IPA and COPD found widely varying sensitivity and specificity—from 18.2% to 100% and 42.8% to 83.5%, respectively. An alternative BDG assay had a sensitivity of 32.4%. Two key caveats in assessing BDG are that most studies utilized multiple samples to derive sensitivity, so the initial sample sensitivity is likely to be lower than reported. Contamination of blood tubes and any plastic reagents can produce false-positive results, as can intravenous immunoglobulin, filtered blood products, hepatic failure, surgical swabs, and hemofiltration.^68^

### Combinations of clinical, radiological, and laboratory features may provide diagnostic confidence for IPA in COPD patients

Overall, a consensus emerged that the combination of compatible radiological features plus 2 laboratory markers was sufficient to make a diagnosis of probable IPA in high-risk exacerbating COPD patients. The 2 laboratory markers might be different tests on the same sample or might be 2 positives of the same test on different samples (even the same day) (eg, 2 positive respiratory cultures or serum GM assays). Characteristic radiological features on CT scan of cavitation or halo sign, plus one positive assay, is also sufficient to diagnose probable IPA, assuming that the time course is relatively short (<3 months) and CPA is not the most likely diagnosis.

**Figure 2:**
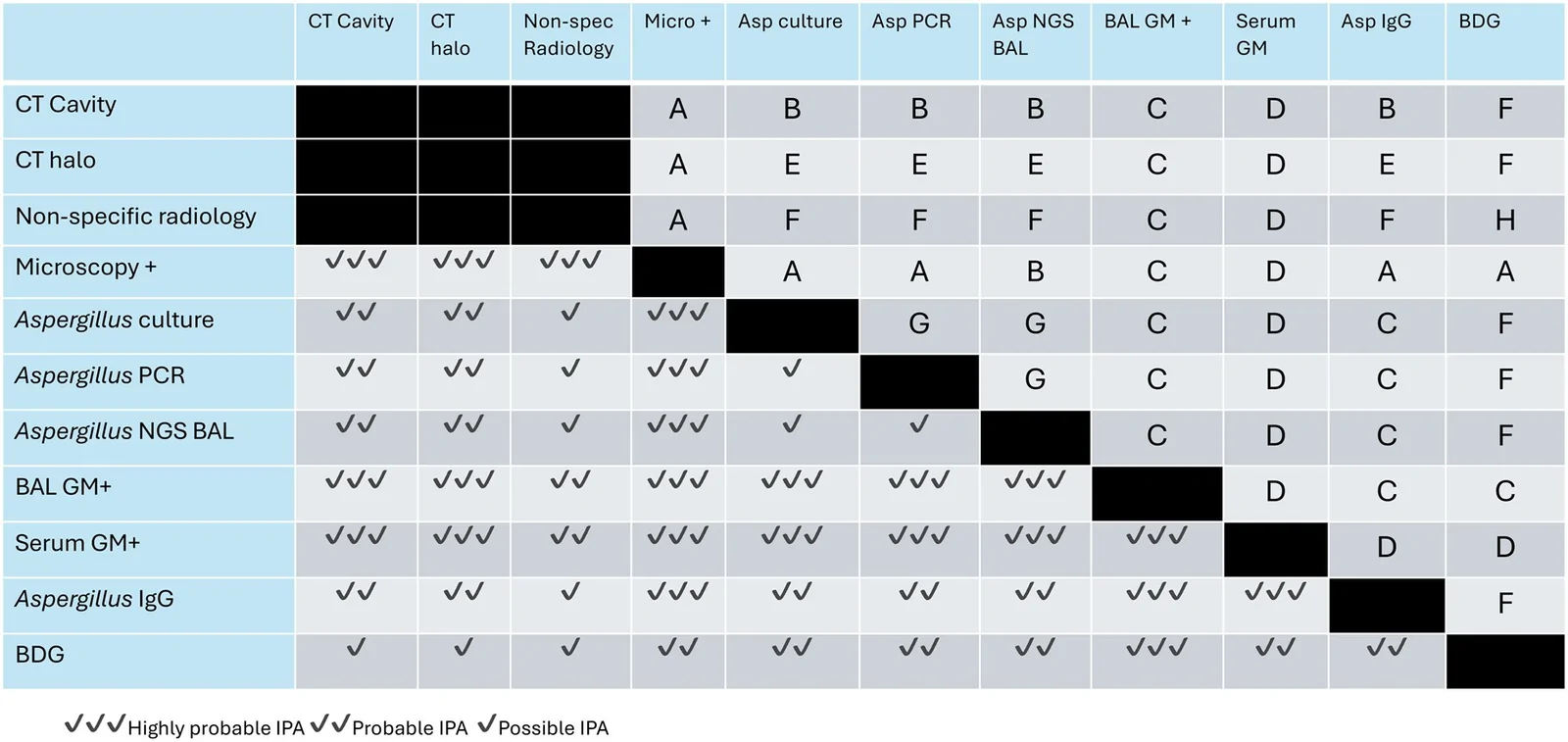
Likelihood matrix for IPA in COPD of combinations of any 2 tests or findings. A: Microscopy positive for hyphae on any respiratory sample provides a high likelihood of *Aspergillus* tracheobronchitis in presence of relevant clinical symptoms and signs. It also suggests a diagnosis of IPA. B: High likelihood of pulmonary aspergillosis, usually IPA, but tracheobronchitis, CPA, or *Aspergillus* bronchitis also possible. C: IPA highly likely (probable) when BAL GM positivity is combined with other *Aspergillus*-specific signs/tests and also considered probable when BAL GM positivity is associated with nonspecific radiology. IPA remains probable when mycological tests indicating the presence of *Aspergillus* (eg, culture, *Aspergillus* IgG, and/or molecular assay) within a sample are positive and are combined with each other or in the presence of lung cavity on chest CT. In addition to IPA, consider subacute invasive aspergillosis or CPA. Positive serum GM assays should be repeated to exclude false-positive results, especially if the ODI is close to the cutoff. D: IPA highly likely (probable) when serum GM positivity is combined with other *Aspergillus*-specific signs/tests and remains probable when supported by nonspecific evidence. To improve diagnostic confidence, confirm the serum result with a second test (as false-positive serum GM results can occur, eg, related to food or antimicrobial agents, blood products), especially if the ODI is close to the cutoff. High and/or persistently elevated serum GM may be a poor prognostic factor. E: IPA highly likely (probable), unless patient also has COVID-19. F: IPA is moderately likely (possible) when the test combination includes at least *Aspergillus*-specific sign/test and the patient has risk factors present and remains moderately likely (possible) if risk factors are present and supported by nonspecific evidence, such as BDG. Other forms of aspergillosis or colonization can occur. G: Confirms the culture is genuine and not a laboratory contaminant; while IPA remains moderately likely (possible), alone this result is not sufficient to diagnose IPA or CPA—needs additional data (imaging and risk factors). H: While a diagnosis of IPA remains moderately likely (possible), a diagnosis of *Pneumocystis* pneumonia should also be considered. Abbreviations: Asp, *Aspergillus*; BDG, 1,3-β-D-glucan; COPD, chronic obstructive pulmonary disease; CPA, chronic pulmonary aspergillosis; CT, computed tomography; GM, galactomannan; IPA, invasive pulmonary aspergillosis; Micro, microscopy; NGS, next-generation sequencing; Non-spec, nonspecific; ODI, optical density index.

Certain combinations of tests make the probability of IPA in COPD even higher, close to certain (Figure 2). However convention has it that documentation of tissue invasion is required for a proven diagnosis of IPA. Such criteria are well laid out by Bulpa et al.^14^ for COPD patients, and for other patients by the revised EORTC/MSGERC criteria.^7^ Unfortunately, such documentation is very unusual, given the fragility of most patients with COPD from a lung biopsy perspective. One possible exception is those with invasive *Aspergillus* tracheobronchitis who undergo bronchoscopy and bronchial biopsy.

Lower levels of diagnostic confidence for IPA in COPD (possible IPA) are also shown in Figure 2. The prime example of such a situation is nonspecific radiological findings and a single positive laboratory test. In hospital settings where specific assays are not done, or have a long turnaround time, possible IPA can be diagnosed, with an empirical treatment decision made, pending further data.

The terms “proven,” “probable,” and “possible” invasive aspergillosis carry specific connotations related to clinical and epidemiological studies and regulatory submissions of novel antifungal agents. “Proven” reflects histological confirmation or pure *Aspergillus* culture from a sterile site (ie, biopsy-obtained). “Probable” relies on biomarkers, culture, and microscopy in a typical at-risk patient and has a >80% likelihood of IPA being the correct diagnosis. “Possible” reflects a high, but lower, likelihood of IPA being the diagnosis, likely >70% certainty. In this document we have used the term “likelihood,” except in Table 3 where we lay out the diagnostic criteria required to establish a probable or possible diagnosis of IPA in COPD patients.

**Table 3:** Modifications of 2007 diagnostic criteria for probable and possible IPA in COPD patients and this workshop.

Type of criteria	**2007 (Bulpa et al.** ^14^ **)**	2026 (workshop)
**Clinical risk factors**	COPD usually treated with steroids and severe according to GOLD (stage III or IV), with recent exacerbation of dyspnea	COPD patient admitted to hospital with an exacerbation and at least 2 risk factors in panels 1-3 from Table 1.
**Radiological features**	Suggestive lesions—pulmonary lesion(s) unresponsive to appropriate antibiotics	Specific CT features of cavitation **OR** halo sign
**Laboratory test**		
** Positive serum biomarkers or tests**	*Aspergillus* IgG **OR** 2× serum GM assays	Serum GM assay^a^ **OR** *Aspergillus* IgG **OR** BDG
** Positive respiratory sample tests**	*Aspergillus* hyphae on direct microscopy **OR** positive *Aspergillus* culture	*Aspergillus* hyphae on direct microscopy **OR** *Aspergillus* culture **OR** PCR **OR** BAL GM antigen **OR** BAL NGS positive for *Aspergillus* DNA
** Probable IPA**	Suggestive radiological lesion(s) **AND** a positive laboratory test	Specific CT imaging **AND** one positive laboratory test (except BDG alone) **OR** nonspecific CT imaging **AND** 2 laboratory tests (see above)
** Possible IPA**	Suggestive lesions without laboratory confirmation	Specific CT imaging without laboratory confirmation **OR** nonspecific CT imaging plus a single positive laboratory test

a Preferably confirmed with a second sample, unless very high.

Abbreviations: BAL, bronchoalveolar lavage fluid sample; BGD, 1,3-β-D-glucan; COPD, chronic obstructive pulmonary disease; CT, computed tomography of the chest; GM, galactomannan; GOLD, Global Initiative for Chronic Obstructive Lung Disease; IPA, invasive pulmonary aspergillosis; NGS, next-generation sequencing detection of *Aspergillus* spp.; PCR, polymerase chain reaction of *Aspergillus* spp.

## Discussion

The incidence of IPA in prospective and retrospective studies in patients admitted to hospital with COPD (almost always an exacerbation, sometimes with bacterial pneumonia or bronchitis) varies from 1 in 910 (0.1%) and 1 in 77 (1.3%) to 1 in 19 (5.3%), with other studies finding approximately 1 in 47 (2.14%) and 1 in 26 (3.9%).^8-12^ The lowest figure (from the United States) was based on an insurance database.^11^ The other studies were primarily based on culture from sputum, with correlative radiological data, sometimes supported by bronchoscopy or serum *Aspergillus* IgG antibody assays. Given the number of patients with COPD admitted to hospital each year (some repeatedly), the incidence of IPA cases globally probably ranges from as low as approximately 750 000 (1.3% of 58 million) to 3 070 000 (5.3%).^69^ The lower number is reflective of the insensitivity of culture, and the higher number possibly linked to active efforts to establish a diagnosis and additional aggravating factors such as prolonged systemic corticosteroid therapy, but both are highly dependent on an uncertain number of admissions to hospital for a COPD exacerbation.

**Figure 3:**
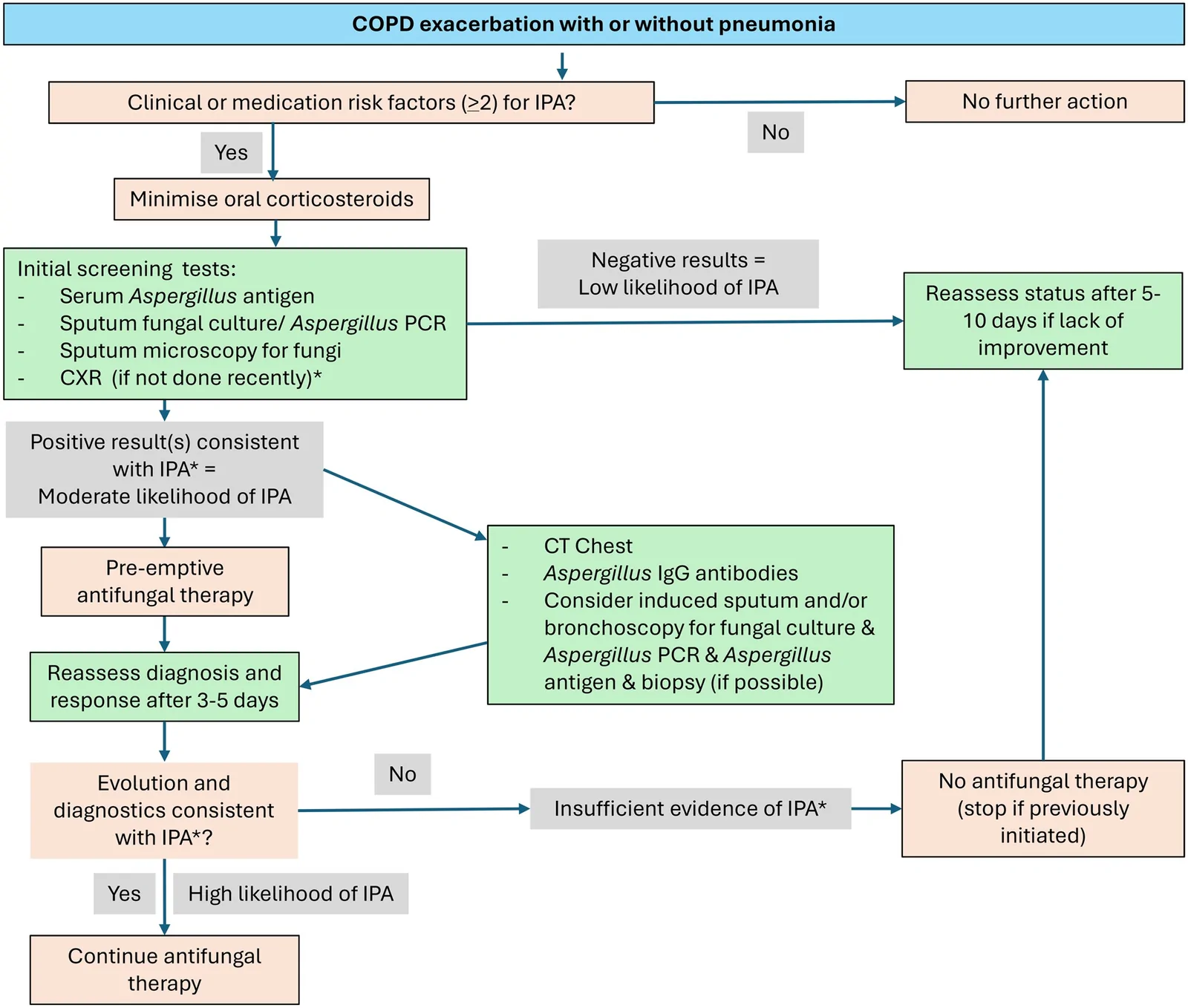
Proposed flow diagram to indicate which COPD patients should be tested for suspected invasive aspergillosis and with which tests. Depending on ease of sample acquisition and turnaround time for testing, preemptive antifungal therapy for invasive aspergillosis may be started, and if tests are negative, then stopped. *IPA should include consideration of invasive *Aspergillus* tracheobronchitis, which can present with persistent wheeze. Early initiation of antifungal therapy for invasive aspergillosis is very important for survival. Abbreviations: COPD, chronic obstructive pulmonary disease; CT, computed tomography; CXR, chest X-ray; IPA, invasive pulmonary aspergillosis.

IPA in its early stages is usually clinically silent, and any symptoms can be masked by corticosteroids. Chest X-rays often do not show new infiltrates. Early diagnosis is crucial for survival from IPA^70^ and plausibly would prevent ICU admission in many patients. The earlier the samples are collected, the greater chance of an early diagnosis and reduction in mortality. This is the basis for the algorithm for early suspicion, diagnostic testing, and preemptive initiation of therapy, with later review once more data are available (Figure 3). This algorithm will need operational research to optimize its utility in different hospital settings. A key aspect of its usage in clinical practice is antifungal stewardship to ensure antifungal therapy is stopped if IPA is not confirmed, for multiple reasons.

Chest X-rays are standard practice for COPD exacerbations requiring hospital admission to exclude differential diagnoses. Gross and late-stage changes of IPA are visible on chest X-rays, but a chest X-ray is much less sensitive than a CT scan, and any new findings are usually attributed to bacterial infection, at least initially. A key goal of our consensus was to identify higher-risk COPD patients requiring early escalation to chest CT, bronchoscopy if possible, or induced sputum and specific testing for aspergillosis. The profile of this patient group includes (1) those with more severe airflow obstruction, those with frequent exacerbations, and those with respiratory failure; (2) those with other comorbid factors such as ischemic heart disease, cardiac failure, or diabetes; and (3) those in receipt of oral corticosteroids (recently or currently), high-dose inhaled steroids, and/or >10 days of antibacterial agents. Less well-defined associations include medium-dose inhaled corticosteroids, bronchiectasis, and emphysematous bullae.

IPA is the most severe and lethal of the disease entities caused by *Aspergillus* spp. in COPD patients. Some patients with IPA complicating COPD have subacute IPA, defined as duration of disease of 1 to 3 months, but this is still usually lethal if not diagnosed, as has been well demonstrated in people with HIV: 83% mortality with a mean time from diagnosis to death of 78 days.^71^ Another common problem is *Aspergillus* colonization, which is itself linked to higher exacerbation frequency.^72^ Whether this is more frequent in those with bronchiectasis complicating COPD is not clear. Colonization may be more common with bronchiectasis, or these patients may have *Aspergillus* bronchitis.^73^^,^^74^ CPA is another entity directly caused by *Aspergillus* spp. in COPD patients, often with additional underlying conditions.^75^ Diagnosed CPA carries a 1-year 16% mortality and a 5-year 35% mortality in those with COPD.^76^ An infrequent subgroup of CPA patients is those with an *Aspergillus* nodule, which is easily misdiagnosed as carcinoma of the lung. An unproven supposition is that high-dose inhaled or oral corticosteroids can precipitate CPA, *Aspergillus* nodule, and IPA in susceptible patients, especially in those colonized. A relatively high frequency of COPD patients are sensitized or allergic to *A. fumigatus*, which is of uncertain clinical importance.^77^

Galactomannan cut-offs were based primarily on the use of the Platelia sandwich ELISA assay (Bio-Rad). More recently, however, other commercial GM and *Aspergillus* antigen tests have become available in different formats (ELISA, lateral flow devices, chemiluminescence-based testing).^78^ The performance characteristics and cut-off values for these assays might be different and should be confirmed by future studies in COPD patients. There is also a caution about how sputum samples are processed, with the standard means of liquefying the sample being with the use of dithiothreitol or heat inactivation, which render the GM test completely negative.^79^^,^^80^

COPD patients have rarely been included in prospective treatment studies of IPA. These studies have usually included those with proven or probable IPA, although initial recruitment of possible IPA with later upgrading (or ruling out) the diagnosis to probable IPA has proven successful in ensuring timely treatment. We are hopeful that COPD patients with IPA will be recruited into treatment studies, allowing a better evidence base for risk profiles and treatment decisions. Given the rising frequency of azole resistance, particularly in *A. fumigatus* and in *A. niger* complex, assessment of new therapies for IPA (such as olorofim or fosmanogepix) will be important in COPD patients, not just classic immunocompromised patients.

Regular and complete implementation of these proposed recommendations will increase the volume of requests for direct microscopy for fungi, fungal culture, GM assays, *Aspergillus* IgG, and probably BDG. In particular, the workflow for respiratory samples may increase the number of samples requiring processing in a category 3 laboratory, because of the concern about *Mycobacterium tuberculosis* or endemic mycoses. However in patients with a COPD exacerbation, once the first sample processed has proven negative for *M. tuberculosis*, subsequent samples could be processed in a category 2 laboratory, if handled in a class 1 cabinet. This could split the work for high-volume cultures, allowing experienced technical staff in mycology to safely handle the samples outside of category 3 laboratory conditions.

We fully recognize that the evidence base underlying this diagnostic consensus is limited in the COPD population specifically, although not for IPA in general. Our presumption, which may be completely or partially incorrect, is that diagnostic performance of laboratory tests for *Aspergillus* spp. on respiratory fluids is transferable from other well-studied populations to the COPD population, and combined with compatible radiological findings in sick patients, should guide their treatment. However, specificity may be lower because of higher rates of airway infection or colonization by *Aspergillus* in COPD. We recognize that indirect tests such as *Aspergillus* IgG antibody and nonspecific assays such as BDG may have less utility, but can at least be used in a supportive manner, in the correct clinical context.

## Supplementary Material

aamag310_Supplementary_Data
