# Supplementary material for: Diagnostic criteria for invasive pulmonary aspergillosis in chronic obstructive pulmonary disease patients
Source: Am J Respir Crit Care Med. 2026 Jul 1;212(9):1913–28. doi: 10.1093/ajrccm/aamag310 (PMC13519330; doi:10.1093/ajrccm/aamag310)
Supplement: Supplementary file 1 [file aamag310_supplementary_data.zip › IPA in AECOPD, supplement final.docx]

Supplementary file

**Diagnostic criteria for invasive pulmonary aspergillosis in COPD patients**

**Denning et al.**

1. Delphi questionnaire results

A. COPD risk assessment

B. Laboratory diagnostics for diagnosing IPA in COPD patients

2. Respiratory sample diagnostics – additional details

A. Fungal culture

B. Detecting galactomannan in sputum

1A. Assessing higher risk in COPD

In terms of risk of invasive aspergillosis in COPD patients, rate the following on a score from 1-7 (7 higher risk, ie from <0.1% to >20% chance of IA when admitted to hospital or presenting in the community with an exacerbation). The purpose of this risk assessment is to provide clinical triggers (or not) for doing specific investigations for IA.

1. Any oral corticosteroid in last 3 months

2. Oral corticosteroid for 5 days in last 3 months

3. Oral corticosteroid for 10 days or more in last 3 months

4. Low dose inhaled corticosteroids

5. High dose inhaled corticosteroid

6. Concurrent major immunocompromising factor (ie leukaemia, neutropenia, transplant)

7. Diabetes

8. Clinically silent bronchiectasis

9. Bronchiectasis with mucus production

10. COPD Gold stage 1

11. COPD Gold stage 2

12. COPD Gold stage 3

13. COPD Gold stage 4

14. Prior exacerbation in last 12 months

15. Home oxygen requirement

16. Ischaemic heart disease

17. Cardiac failure

18. Treatment for lung cancer

19. Severe emphysema

20. Large bullae

Responses (n=18):

| **Rank** | **Question** | **Mean** | **SD** | **min_rating** | **max_rating** |
| --- | --- | --- | --- | --- | --- |
| 1 | Concurrent major immunocompromising factor (ie leukaemia, neutropenia, transplant) | 6.56 | 0.51 | 6 | 7 |
| 2 | Oral corticosteroid for 10 days or more in last 3 months | 5.72 | 1.07 | 4 | 7 |
| 3 | COPD Gold stage 4 | 5.11 | 1.13 | 3 | 7 |
| 4 | Any oral corticosteroid in last 3 months | 5.11 | 1.15 | 3 | 7 |
| 5 | Severe emphysema | 5.00 | 1.08 | 3 | 7 |
| 6 | Treatment for lung cancer | 4.83 | 1.10 | 3 | 6 |
| 7 | Oral corticosteroid for 5 or more days in last 3 months | 4.63 | 1.71 | 1 | 7 |
| 8 | Home oxygen requirement | 4.56 | 1.42 | 2 | 7 |
| 9 | Diabetes | 4.44 | 1.42 | 1 | 6 |
| 10 | Large bullae | 4.28 | 1.45 | 2 | 6 |
| 11 | High dose inhaled corticosteroid | 4.11 | 1.18 | 2 | 7 |
| 12 | COPD Gold stage 3 | 3.89 | 1.28 | 1 | 6 |
| 13 | Bronchiectasis with mucus production | 3.83 | 1.47 | 1 | 6 |
| 14 | Prior exacerbation in last 12 months | 3.78 | 1.26 | 1 | 6 |
| 15 | Cardiac failure | 3.56 | 1.38 | 2 | 6 |
| 16 | Ischaemic heart disease | 3.44 | 1.50 | 1 | 5 |
| 17 | Clinically silent bronchiectasis | 2.94 | 1.66 | 1 | 6 |
| 18 | COPD Gold stage 2 | 2.06 | 1.11 | 1 | 5 |
| 19 | Low dose inhaled corticosteroids | 2.06 | 1.35 | 1 | 6 |
| 20 | COPD Gold stage 1 | 1.56 | 0.70 | 1 | 3 |

1B. Laboratory testing for diagnosing IPA in COPD patients

Performance of diagnostic/biomarker assays for IA in COPD patients

In terms of test value for diagnosing invasive aspergillosis in COPD patients, rate the following on a score from 1-7 (7 excellent performance (combination of sensitivity and specificity)).

1. Serum galactomannan

2. Sputum galactomannan

3. Serum/blood *Aspergillus* PCR

4. Sputum PCR

5. Serum beta 1,3-D-glucan (BDG)

6. Serum *Aspergillus* IgG

7. Sputum bacterial culture (to grow *Aspergillus*)

8. Sputum standard fungal culture

9. Sputum high volume fungal culture

10. Sputum direct microscopy for fungi

11. Sputum Next Generation Sequencing (NGS) for fungi

12. BAL galactomannan

13. Bronchoscopy sample fungal culture

14. Bronchoscopy sample direct microscopy for fungi

15. Bronchoscopy PCR

16. Bronchoscopy sample NGS for fungi

Responses (n=19):

| **Section rank** | **Overall rank** | **Question** | **Mean** | **SD** | **min_rating** | **max_rating** |
| --- | --- | --- | --- | --- | --- | --- |
| 1 | 5 | BAL galactomannan | 5.7 | 0.87 | 4 | 7 |
| 2 | 9 | Bronchoscopy sample fungal culture | 5.3 | 0.95 | 3 | 6 |
| 3 | 13 | Bronchoscopy PCR | 5.1 | 0.85 | 3 | 6 |
| 4 | 14 | Bronchoscopy sample direct microscopy for fungi | 5.1 | 1.43 | 2 | 7 |
| 5 | 18 | Sputum high volume fungal culture | 4.8 | 1.07 | 2 | 6 |
| 6 | 23 | Sputum direct microscopy for fungi | 4.5 | 1.50 | 2 | 6 |
| 7 | 24 | Bronchoscopy sample NGS for fungi | 4.4 | 1.12 | 2 | 6 |
| 8 | 28 | Sputum standard fungal culture | 4.2 | 1.23 | 2 | 4 |
| 9 | 29 | Sputum bacterial culture (to grow *Aspergillus*) | 3.9 | 1.18 | 2 | 6 |
| 10 | 34 | Serum/blood Aspergillus PCR | 3.8 | 1.72 | 1 | 6 |
| 11 | 30 | Sputum PCR | 3.8 | 1.50 | 1 | 5 |
| 12 | 35 | Serum Aspergillus IgG | 3.7 | 1.14 | 2 | 6 |
| 13 | 36 | Serum galactomannan | 3.7 | 1.63 | 1 | 7 |
| 14 | 38 | Serum GM | 3.6 | 1.46 | 1 | 6 |
| 15 | 40 | Sputum Next Generation Sequencing (NGS) for fungi | 3.5 | 1.31 | 1 | 6 |
| 16 | 42 | Serum beta 1,3-D-glucan (BDG) | 3.2 | 1.30 | 1 | 6 |
| 17 | 43 | Sputum GM | 2.5 | 0.96 | 1 | 5 |

Combinations of tests

The next exercise addresses selective combinations of tests. Rate the confidence of a diagnosis of IA using these tests, in a COPD patient with a current exacerbation and infiltrates in the chest. Rate the likelihood of IA on a score from 1-7 (1 very unlikely; 7 highly likely). [As a general statement a probability of IA of <10% is unlikely or very unlikely and >70% very or highly likely]. For this exercise, assume different commercial assays of the same test are approximately equivalent.

A. Both tests positive

17. Sputum culture and serum GM

18. Sputum culture and *Aspergillus* IgG

19. Sputum culture and BDG

20. Sputum PCR and serum GM

21. Sputum microscopy and *Aspergillus* IgG

22. Bronchoscopy sample GM and culture

23. Bronchoscopy sample GM and serum GM

24. Bronchoscopy sample fungal culture and serum *Aspergillus* IgG

25. Bronchoscopy sample fungal culture and serum BDG

Responses (n=19):

| **Section rank** | **Overall rank** | **Question** | **Mean** | **SD** | **min_rating** | **max_rating** |
| --- | --- | --- | --- | --- | --- | --- |
| 2 | 2 | A. Both tests positive Bronchoscopy sample GM and culture | 5.9 | 0.88 | 4 | 7 |
| 2 | 1 | A. Both tests positive Bronchoscopy sample GM and serum GM | 5.9 | 0.97 | 4 | 7 |
| 3 | 6 | A. Both tests positive Sputum culture and serum GM | 5.7 | 0.82 | 4 | 7 |
| 4 | 7 | A. Both tests positive Sputum PCR and serum GM | 5.5 | 1.12 | 3 | 7 |
| 5 | 10 | A. Both tests positive Bronchoscopy sample fungal culture and serum Aspergillus IgG | 5.2 | 0.92 | 4 | 7 |
| 6 | 12 | A. Both tests positive Bronchoscopy sample fungal culture and serum BDG | 5.2 | 0.60 | 4 | 7 |
| 7 | 17 | A. Both tests positive Sputum culture and Aspergillus IgG | 4.8 | 1.01 | 3 | 7 |
| 8 | 19 | A. Both tests positive Sputum microscopy and Aspergillus IgG | 4.7 | 1.37 | 2 | 7 |
| 9 | 26 | A. Both tests positive Sputum culture and BDG | 4.3 | 0.79 | 3 | 7 |

B. One test positive, one test negative

26. Sputum culture or PCR negative, serum GM positive

27. Sputum culture or PCR positive, serum GM negative

28. Sputum culture or PCR negative, serum *Aspergillus* IgG positive

29. Sputum culture or PCR positive, serum *Aspergillus* IgG negative

30. Sputum culture negative, serum BDG positive

31. Sputum culture positive, serum BDG negative

32. Bronchoscopy GM positive, serum GM negative

33. Bronchoscopy sample GM negative and bronchoscopy sample culture positive

34. Bronchoscopy sample GM negative and bronchoscopy sample PCR positive

35. Bronchoscopy sample GM positive and serum Aspergillus IgG negative

Responses (n=19):

| **Section rank** | **Overall rank** | **Question** | **Mean** | **SD** | **min_rating** | **max_rating** |
| --- | --- | --- | --- | --- | --- | --- |
| 1 | 16 | B. One test positive, one test negative Bronchoscopy GM positive, serum GM negative | 4.9 | 1.08 | 3 | 7 |
| 2 | 20 | B. One test positive, one test negative Bronchoscopy sample GM positive and serum Aspergillus IgG negative | 4.7 | 0.96 | 3 | 7 |
| 3 | 22 | B. One test positive, one test negative Sputum culture or PCR negative, serum GM positive | 4.5 | 1.22 | 2 | 7 |
| 4 | 25 | B. One test positive, one test negative Bronchoscopy sample GM negative and bronchoscopy sample culture positive | 4.3 | 1.14 | 2 | 6 |
| 5 | 27 | B. One test positive, one test negative Bronchoscopy sample GM negative and bronchoscopy sample PCR positive | 4.2 | 1.06 | 2 | 6 |
| 6 | 31 | B. One test positive, one test negative Sputum culture or PCR positive, serum Aspergillus IgG negative | 3.8 | 1.23 | 2 | 6 |
| 7 | 32 | B. One test positive, one test negative Sputum culture or PCR positive, serum GM negative | 3.8 | 0.98 | 2 | 7 |
| 8 | 33 | B. One test positive, one test negative Sputum culture positive, serum BDG negative | 3.8 | 1.13 | 1 | 7 |
| 9 | 39 | B. One test positive, one test negative Sputum culture or PCR negative, serum Aspergillus IgG positive | 3.5 | 1.17 | 2 | 7 |
| 10 | 41 | B. One test positive, one test negative Sputum culture negative, serum BDG positive | 3.4 | 1.54 | 1 | 7 |

Workload/cost versus performance uncertainty

There is a cost-benefit uncertainty for laboratories, in terms of workload and yield from individual tests. The following questions are designed to address this, specifically in COPD patients admitted to hospital and suspected to have IPA. Rate the likelihood of IA on a score from 1-7 (1 very unlikely; 7 highly likely).

36. Should all sputum samples from COPD patients suspected of having IPA be examined for fungi using direct microscopy?

37. Should all sputum samples from COPD patients be cultured on fungal media?

38. If AFB smear or other rapid test for TB is negative, can high volume fungal culture be done in a Category 2 laboratory (ie not Category 3)?

39. If a chest X-ray shows no sign of cavitation, can high volume fungal culture be done in a category 2 laboratory (ie not Category 3)?

40. Should all serum samples from COPD patients suspected of having IPA be tested for GM?

41. Should all serum samples from COPD patients suspected of having IPA be tested for Aspergillus IgG?

42. Should all serum samples from COPD patients suspected of having IPA be tested for BDG?

Responses (n=19):

| **Rank** | **Question** | **Mean** | **SD** | **min_rating** | **max_rating** |
| --- | --- | --- | --- | --- | --- |
| 1 | If AFB smear or other rapid test for TB is negative, can high volume fungal culture be done in a Category 2 laboratory (ie not Category 3)? | 5.9 | 1.02 | 4 | 6 |
| 2 | Should all sputum samples from COPD patients suspected of having IPA be examined for fungi using direct microscopy? | 5.8 | 1.31 | 3 | 6 |
| 3 | Should all sputum samples from COPD patients be cultured on fungal media? | 5.4 | 1.58 | 2 | 5 |
| 4 | Should all serum samples from COPD patients suspected of having IPA be tested for GM? | 5.2 | 1.50 | 2 | 6 |
| 5 | If a chest X-ray shows no sign of cavitation, can high volume fungal culture be done in a category 2 laboratory (ie not Category 3)? | 5.0 | 1.50 | 2 | 6 |
| 6 | Should all serum samples from COPD patients suspected of having IPA be tested for Aspergillus IgG? | 4.6 | 1.65 | 2 | 5 |
| 7 | Should all serum samples from COPD patients suspected of having IPA be tested for BDG? | 3.7 | 1.45 | 1 | 6 |

2A. *Fungal culture*

Fungal culture is neither sensitive nor specific for IPA in COPD. Sputum should be submitted with a request for ‘fungal culture’ at a minimum, recognising that a request for ‘culture’ will usually be processed only for bacterial culture. High volume culture showed a 3-5-fold higher yield in other contexts (ABPA, CPA, COVID-19 related IPA) (CAPA) (Vergidis, 2018; Jabeen, 2020; van Haren, 2026).

Many laboratories require all respiratory samples to pass through a Category 3 (Cat3) laboratory first, and then for the sputum to be deactivated before leaving the laboratory, because of perceived risk of tuberculosis acquisition by laboratory personnel. All respiratory samples should be processed in a Class 1 (or Class 2) hood anyway, reducing exposure risk for all pathogens to the laboratory staff.

There are several workarounds for sample processing outside a Cat3 laboratory, although this could incur delays: 1) Process samples outside Cat3 when prior samples have been shown to be negative for mycobacteria by microscopy and/or molecular assays; 2) in low burden TB settings, undertake a rapid risk assessment based on the lack of cavitary features on imaging (and other typical clinical features of infectious TB), and the devastating consequences to patients of missing (or delaying) a diagnosis of IPA.

Processing multiple specimens for culture increases diagnostic sensitivity, for pulmonary aspergillosis (McCarthy, 1971; Chakrabarti, 2002, Katriina, 2023; van Haren, 2026), although not assessed in COPD patients specifically.

2B. *Aspergillus* antigen (GM) on sputum

International consensus definitions have recently been published recommending GM as a mycological criterion for diagnosis of IPA in patients in intensive care (ICU), including cases of moderate/ severe COPD, who have compatible clinical signs and symptoms (Bassetti et al, 2024; Lamoth, 2025). They identified optical density index (ODI) cut-offs (ODI) of ≥1, and >0.5 as thresholds for bronchoalveolar lavage fluid (BALF) and serum positivity, respectively. These cut-offs were based primarily on the use of the Platelia *Aspergillus* Ag assay (BioRad). Higher ODI values are typically associated with improved specificity and diagnostic confidence.

Few studies have evaluated GM detection in sputum samples. A retrospective analysis of routinely collected sputum GM values from 218 patients with idiopathic bronchiectasis and COPD as the main underlying diseases showed a sensitivity of 77% and specificity of 78% for the diagnosis of CPA (GM was evaluated against *Aspergillus* culture/IgG). There was a strong overlap with the sputum GM values of the colonisation group (Nuh, 2022). The value of sputum GM detection (using the Dynamiker *Aspergillus* GM assay) for the diagnosis of CPA was also evaluated in COPD patients referred to two pulmonary disease reference centres in Iran. At the optimal cutoff value (ODI = 1.44) sensitivity and specificity were found to be 83.3% and 74.2% respectively. In a study of *Aspergillus* detection and risk factors in patients with COPD and bronchiectasis, induced sputum GM ODIs were significantly higher in patients with COPD and bronchiectasis (n= 17) compared with patients with COPD alone (n=18) and ranged between the levels of healthy controls and *A. fumigatus*-colonized cystic fibrosis patients (Everaerts et al. 2018). Of note, 3 of 7 induced sputum samples from healthy controls had GM ODI values >1.5. In a prospective diagnostic study for invasive aspergillosis in patients with underlying respiratory disease, induced sputum-based tests were evaluated in a case-control subcohort (n=127, 72 with COPD). Sputum GM (≥2 ODI) had similar sensitivity (84%) and slightly lower specificity (87%) than BAL GM (≥1 ODI) (Xiao. 2022). Best performance of GM on proximal airway samples (induced sputum or tracheal aspirates) in a retrospective study in cancer patients (70 proven/probable IPA cases) was obtained with an ODI cutoff of ≥0.725, resulting in a sensitivity of 89.7% and specificity 91.5% leading to the conclusion that performance of the GM test on non-invasive proximal airway samples is comparable or even better than those from serum and BAL (Jhun, 2024). Important to note is that of the 624 patients in whom GM test was performed, 312 had a positive test (50%) but patients with saprophytic or colonizing syndromes of *Aspergillus* (n=17) and patients with non-specific CT findings (n=115) were excluded from the analysis (Chun JY et al. 2024). Thus, the performance characteristics presented in the study do only apply for cancer patients meeting the radiological CT criteria as defined by the EORTC/MSGERC). In a mixed patient population, Chia-Ta Tsai (2026) concluded that non-induced sputum GM demonstrated moderate diagnostic performance relative to bronchoscopic mycological findings. In this patient population (n=1002), the overall positivity rate of sputum GM was 51% which suggests that the detection of *Aspergillus* antigens in the airway is common among hospitalised patients. Nevertheless, they observed a significant linear trend between increasing sputum GM index values and composite bronchoscopic mycological positivity (Tsai CT et al. 2026)

Sputum samples need to be diluted/liquified to make them suitable for pipetting and thus for GM analysis. In the study by Nuh et al. sputum was diluted with an equal volume of phosphate buffered saline, which is preferable to the use of dithiothreitol (Sputasol®) (Everaerts, 2018). Heating samples to allow movement out of a Cat3 laboratory destroys GM.

References
Vergidis P, Moore CB, Novak-Frazer L, Richardson R, Walker A, Denning DW, et al. High-volume culture and quantitative Real-Time PCR for the detection of *Aspergillus* in sputum. Clin Microbiol Infect 2020;26:935-940.

Jabeen K, Farooqi J, Irfan M, Ali SA, Denning DW. Diagnostic dilemma in COVID-19-associated pulmonary aspergillosis. Lancet Infect Dis. 2021 Jun;21(6):767. doi: 10.1016/S1473-3099(21)00066-9.

van Haren MHI, Michon L, Verweij PE, Reijers MHE, Brüggemann RJ, van Mook S, van der Lee H, Melchers WJG, Pas SD, Buil JB. High-Volume Culture Improves Diagnostic Yield of *Aspergillus* Species from Sputum. Mycoses 2026 Mar;69(3):e70168. doi: 10.1111/myc.70168.

McCarthy DS, Pepys J. Allergic bronchopulmonary aspergillosis. Clinical immunology. 2. Skin, nasal and bronchial tests. Clin Allergy 1971; 1:415–32.

Chakrabarti A, Sethi S, Raman DS, Behera D. Eight-year study of allergic broncho-pulmonary aspergillosis in an Indian teaching hospital. Mycoses 2002; 45:295–9.

Katriina P, Veli-Jukka A, Ulla H. The clinical significance of *Aspergillus*-positive respiratory samples. Mycoses 2023 May;66(5):387-395. doi: 10.1111/myc.13566.

Bassetti M, Giacobbe DR, Agvald-Ohman C, Akova M, Alastruey-Izquierdo A, Arikan-Akdagli S et al. Invasive Fungal Diseases in Adult Patients in Intensive Care Unit (FUNDICU): 2024 consensus definitions from ESGCIP, EFISG, ESICM, ECMM, MSGERC, ISAC, and ISHAM. Intensive Care Med. 2024 Apr;50(4):502-515. doi: 10.1007/s00134-024-07341-7.

Lamoth F, Albrich WC, Ragozzino S, Bosetti D, Delaloye J, El Khoury C,et al; Fungal Infection Network of Switzerland (FUNGINOS). Management of Invasive Pulmonary Aspergillosis in Intensive Care Units: Guidelines From the Fungal Infection Network of Switzerland (FUNGINOS). Mycoses. 2025 Nov;68(11):e70132. doi: 10.1111/myc.70132.

Nuh A, Ramadan N, Shah A, Armstrong-James D. Sputum Galactomannan Has Utility in the Diagnosis of Chronic Pulmonary Aspergillosis. J Fungi (Basel) 2022 Feb 14;8(2):188. doi: 10.3390/jof8020188

Everaerts S, Lagrou K, Vermeersch K, Dupont LJ, Vanaudenaerde BM, Janssens W. *Aspergillus fumigatus* Detection and Risk Factors in Patients with COPD-Bronchiectasis Overlap. Int J Mol Sci. 2018 Feb 9;19(2):523. doi: 10.3390/ijms19020523.

Xiao W, Du L, Cai L, Miao T, Mao B, Wen F, Gibson PG, Gong D, Zeng Y, Kang M, Du X, Qu J, Wang Y, Liu X, Feng R, Fu Existing tests vs. novel non-invasive assays for detection of invasive aspergillosis in patients with respiratory diseases. Chin Med J (Engl). 2022 Jul 5;135(13):1545-1554.

Chun JY, Jeong SJ, Kim S, Choi S, Lee JH, Chung HS, Park S, Lee H, Kim HY, Hwangbo B, Choi YJ.J Performance of the galactomannan test for the diagnosis of invasive pulmonary aspergillosis using non-invasive proximal airway samples. Infect. 2024 Jun;88(6):106159.

Tsai CT, Liang SY, Lai CH, Tsai YN, Chiu CT, Huang CK. Clinical utility of non-induced sputum galactomannan testing for suspected invasive pulmonary aspergillosis: a real-world retrospective cohort study. Infection. 2026 Mar 28. doi: 10.1007/s15010-026-02784-0.
